# Supplementary material for: Standard melanoma-associated markers do not identify the MM127 metastatic melanoma cell line
Source: Sci Rep. 2016 Apr 18;6:24569. doi: 10.1038/srep24569 (PMC4834532; doi:10.1038/srep24569)
Supplement: Supplementary Information [file srep24569-s1.pdf]

Supplementary Material

Standard melanoma-associated markers do not identify the MM127 metastatic melanoma cell line.

Parvathi Haridas[1,2], Jacqui A. McGovern[1], Abhishek S. Kashyap[1], D. L. Sean McElwain[1,2] and Matthew J. Simpson[1,2,*]

[1]Institute of Health and Biomedical Innovation, Queensland University of Technology (QUT), Kelvin Grove 4059, Australia.

[2]School of Mathematical Sciences, QUT, PO Box 2434, Brisbane 4001, Australia.

*Corresponding Author: matthew.simpson@qut.edu.au

Table of Contents

Additional Results: Immunofluorescence ……………………………………………..2
Additional Results: qRT-PCR ……………………………………………..3
DSMZ database matching: WM35 ……………………………………………..5
DSMZ database matching: WM793 ……………………………………………..6
DSMZ database matching: SK-MEL-28 ……………………………………………..7
DSMZ database matching: MM127 ……………………………………………..8

Immunofluorescence

Results for the negative control (HaCaT) confirm that the HaCaT cell line is negative for S100, HMB-45 and Melan-A [Fig. S1].

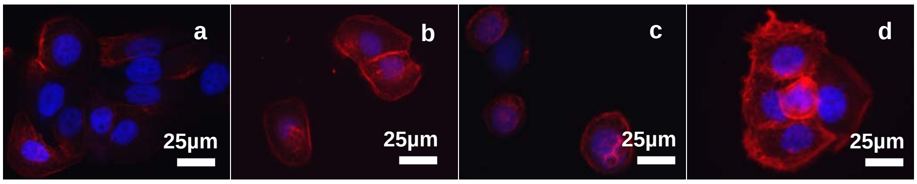

Figure S1: Immunofluorescence analysis for the negative control (HaCaT) do not express melanoma-associated proteins. Cells are fixed in 4% paraformaldehyde and stained for (a) S100 (green), (b) HMB-45 (green) and (c) Melan-A (green). The nucleus (blue) and f-actin (red) are highlighted. (d) Shows results for the secondary antibody only.

Additional results confirm that some of the primary cells we consider (fibroblasts and keratinocytes) are also negative for S100, HMB-45 and Melan-A. Alternatively, primary melanocytes are positive for all the three melanoma-associated markers. As a result, we do not use primary keratinocytes and fibroblasts as negative controls as they are always at risk of being contaminated with melanocytes, which could lead to false positive results. Therefore, we use the HaCaT cell line as the negative control.

Quantitative reverse transcription-polymerase chain reaction (qRT-PCR)

Total cellular RNA is extracted from melanoma cell lines (WM35, WM793, MM127, SK-MEL-28), and the non-melanoma control cell line (HaCaT) using Trizol Reagent (Life Technologies) following the manufacturer's protocol. One µg of RNA is reverse transcribed and cDNA is synthesised using SuperScript® III First-Strand (Life Technologies). The gene-specific primers (Sigma Aldrich) used in qRT-PCR are given in Table S1.

Table S1: Gene and primer sequences.

Gene Symbol	Primer Sequence	Gene Bank Accession Number
MLANA	Forward:5' CACGGCCACTCTTACACCAC 3' Reverse: 5' GGAGCATTGGGAACCACAGG 3'	NM_006272.2
S100B	Forward 5' TGGCCCTCATCGACGTTTTC 3' Reverse 5' ATGTTCAAAGAACTCGTGGCA 3'	NM_005511.1
PMEL	Forward 5' AGTTCTAGGGGGCCCAGTGTCT Reverse 5' GGGCCAGGCTCCAGGTAAGTAT 3'	NM_006928.4
MITF	Forward 5' CATTGTTATGCTGGAAATGCTAGAA Reverse 5' GGCTTGCTGTATGTGGTACTTGG 3'	NM_198178.2
TYR	Forward 5' GGCTGTTTTGTACTGCCTGCT 3' Reverse 5' AGGAGACACAGGCTCTAGGGAA 3'	NM_000372.4
RPL32	Forward: 5' CCCCTTGTGAAGCCCAAGA 3' Reverse: 5' GACTGGTGCCGGATGAACTT 3'	NM_001007073.1

The expression of *MITF* and *TYR* in the MM127 cell line is very similar to the negative control [Fig. S2].

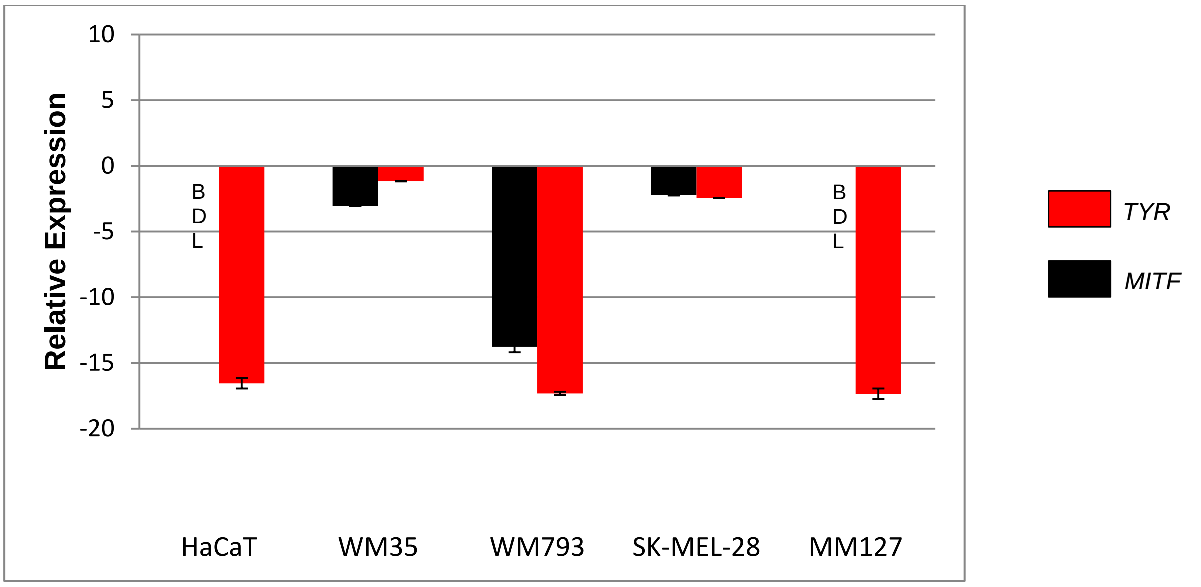

Figure S2: Gene expression of *MITF* and *TYR* relative to *RPL32*. Results for *TYR* and *MITF* in the HaCaT and MM127 cell lines are below the detectable limit (BDL). qRT-PCR results are reported as the difference between the expression of melanoma-associated genes for the negative control (HaCaT) and various cell lines. Values correspond to the mean ΔCt (n=3), where ΔCt = Ct (*RPL32*) – Ct (*target gene*). Error bars indicate the standard error (n=3).

Result of STR matching analysis by your data.

- DSMZ Profile Database -

A graphical presentation is shown at the bottom of this page.

EV	Cell No.	Cell name	Locus names									Figures
			D5S818	D13S317	D7S820	D16S539	VWA	TH01	AM	TPOX	CSF1PO	
	Query (Your Cell)		*11,12*	*11*	*10,13*	*12,13*	*17*	*9.3*	*x*	*8*	*10*	
1.20(36/30)	CRL-2807	WM35	11,12	11,11	10,13	12,13	17,17	9.3,9.3	X,X	8,8	10,10	-
1.07(32/30)	196	COLO-699	11,12	11,11	11,13	12,12	17,17	9,9.3	X,X	8,8	10,10	-
1.00(30/30)	503	HCC-1599	12,12	11,11	10,11	12,13	17,17	9.3,9.3	X,X	8,8	12,12	-
1.00(30/30)	CRL-10302	SW756	11,12	11,11	10,12	12,13	17,17	9.3,9.3	X,X	8,8	11,12	-
1.00(30/30)	CRL-2331	HCC1599	12,12	11,11	10,11	12,13	17,17	9.3,9.3	X,X	8,8	12,12	-
1.00(30/30)	CRL-9446	CHL-1	11,12	8,11	11,13	13,13	17,17	9,9.3	X,X	8,8	10,10	-
1.00(30/30)	CRL-9451	CHL-2 pPA003	11,12	8,11	11,13	13,13	17,17	9,9.3	X,X	8,8	10,10	-
1.00(30/30)	CRL-9607	HMCB	11,12	8,11	11,13	13,13	17,17	9,9.3	X,X	8,8	10,10	-
0.93(28/30)	544	H-2171	13,13	11,11	11,13	12,13	17,17	9.3,9.3	X,Y	8,8	10,10	-
0.93(28/30)	734	Calu-6	11,11	11,11	10,10	13,13	17,17	9,9	X,X	8,8	12,12	-
0.93(28/30)	CRL-2332	HCC1599 BL	12,12	8,11	10,11	12,13	17,17	9.3,9.3	X,X	8,8	12,13	-
0.93(28/30)	HTB-126	Hs 578T	11,11	11,11	10,10	9,12	17,17	9,9.3	X,X	8,8	13,13	-
0.93(28/30)	HTB-183	NCI-H661 [H661]	11,11	11,11	8,10	12,12	17,17	8,8	X,Y	8,8	10,10	-

Result of STR matching analysis by your data.

- DSMZ Profile Database -

A graphical presentation is shown at the bottom of this page.

EV	Cell No.	Cell name	D5S818	D13S317	D7S820	D16S539	VWA	TH01	AM	TPOX	CSF1PO	Figures
		Query (Your Cell)	*10,12*	*8,14*	*10,12*	*12*	*17*	*6,8*	*x,y*	*8*	*11*	
1.12(36/32)	CRL-2806	WM793B [Part of the Wistar Special Collection]	10,12	8,14	10,12	12,12	17,17	6,8	X,Y	8,8	11,11	-
1.06(34/32)	CRL-2812	1205Lu [Part of the Wistar Special Collection]	10,12	8,14	10,12	12,12	17,17	6,8	X,Y	8,9	11,11	-
0.88(28/32)	518	SK-ES-1	12,12	8,9	10,11	12,12	14,17	6,9.3	X,Y	8,8	11,11	-
0.88(28/32)	546	SEM	12,12	14,14	10,12	8,10	17,17	6,9.3	X,X	8,8	11,12	-
0.88(28/32)	CRL-2708	CCD-1139Sk	12,12	9,14	10,12	11,12	14,17	6,6	X,Y	8,8	11,12	-
0.81(26/32)	566	HCC-827	12,12	9,9	11,12	12,12	18,18	6,6	X,X	8,8	11,11	-
0.81(26/32)	CRL-5807	NCI-H358 [H-358, H358]	10,12	8,12	10,11	12,13	17,17	6,6	X,Y	8,9	11,12	-
0.81(26/32)	CRL-5902	NCI-H1876 [H1876]	11,11	12,12	10,12	12,12	17,17	6,6	X,Y	8,9	11,11	-
0.81(26/32)	CRL-5903	NCI-H1882 [H1882]	11,11	12,12	10,12	12,12	17,17	6,6	X,Y	8,9	11,11	-
0.81(26/32)	CRL-5927	NCI-H2141 [H2141]	12,12	14,14	10,12	8,8	19,19	6,8	X,Y	8,9	11,11	-
0.81(26/32)	RCB0662	HOTHC	12,12	8,8	8,8	12,12	14,16	6,6	X,X	8,8	10,11	-
0.81(26/32)	RCB0690	HOTHC-SF	12,12	8,8	8,8	12,12	14,16	6,6	X,X	8,8	10,11	-
0.81(26/32)	CRL-2868	HCC827	12,12	9,9	11,12	12,12	18,18	6,6	X,X	8,8	11,11	-

Result of STR matching analysis by your data.

- DSMZ Profile Database -

A graphical presentation is shown at the bottom of this page.

EV	Cell No.	Cell name	Locus names									Figures
			D5S818	D13S317	D7S820	D16S539	VWA	TH01	AM	TPOX	CSF1PO	
	Query (Your Cell)		*11,13*	*11,12*	*10*	*9,12*	*16,19*	*7*	*x,y*	*8,12*	*10,12*	
1.06(36/34)	HTB-72	SK-MEL-28	11,13	11,12	10,10	9,12	16,19	7,7	X,Y	8,12	10,12	-
1.06(36/34)	RCB1930	SK-MEL-28	11,13	11,12	10,10	9,12	16,19	7,7	X,Y	8,12	10,12	-
0.94(32/34)	JCRB0820	EBC-1	11,11	12,12	10,11	9,9	16,17	7,7	X,X	8,8	10,10	-
0.94(32/34)	CRL-2056	CCD-1043Sk	13,13	11,12	10,10	9,11	16,19	7,7	X,Y	8,11	12,12	-
0.88(30/34)	17	HDLM-2	11,11	11,12	8,10	12,12	19,19	6,7	X,Y	8,8	11,12	-
0.88(30/34)	RCB1678	JHOS-4	11,11	11,11	10,10	9,10	14,14	7,7	X,X	8,12	10,10	-
0.88(30/34)	RCB1941	Li-7	9,11	11,11	10,11	9,9	16,19	7,7	X,X	8,11	12,12	-
0.88(30/34)	RCB1965	EBC-1	11,11	12,12	10,11	8,9	16,17	7,7	X,X	8,8	10,10	-
0.88(30/34)	RCB1966	S1	11,11	11,12	8,11	12,12	16,16	7,7	X,X	8,11	12,12	-
0.82(28/34)	157	MHH-NB-11	11,11	12,12	8,9	9,13	16,16	7,9.3	X,Y	8,8	12,12	-
0.82(28/34)	207	NB-4	13,13	11,12	10,13	9,9	16,19	7,9.3	X,X	8,11	11,12	-
0.82(28/34)	402	RPMI-8226	11,13	11,11	9,10	9,9	16,16	8,8	X,Y	8,11	12,12	-
0.82(28/34)	CRL-1583	3A(tPA-30-1)	11,13	11,11	10,10	9,12	16,17	9,9.3	X,X	8,11	10,12	-

Result of STR matching analysis by your data.

- DSMZ Profile Database -

A graphical presentation is shown at the bottom of this page.

EV	Cell No.	Cell name	D5S818	D13S317	D7S820	D16S539	VWA	TH01	AM	TPOX	CSF1PO	Figures
	Query (Your Cell)		*11,12*	*12,13*	*10,12*	*10,13*	*16,18*	*8,9*	*x,y*	*11,12*	*10,11*	
0.89(32/36)	680	NALM-16	11,11	12,12	10,10	13,13	17,17	8,8	X,X	11,11	11,11	-
0.83(30/36)	431	42-MG-BA	11,12	13,13	10,10	10,10	18,18	6,8	X,X	8,11	10,12	-
0.83(30/36)	517	HCC-1143	11,11	12,12	12,12	11,13	16,16	9.3,9.3	X,X	12,12	10,10	-
0.83(30/36)	CRL-2321	HCC1143	11,11	12,12	12,12	11,13	16,16	9.3,9.3	X,X	12,12	10,10	-
0.83(30/36)	CRL-5845	NCI-H841 [H841]	11,12	12,12	10,13	13,13	16,17	6,9	X,Y	11,12	11,11	-
0.83(30/36)	HTB-73	SK-MEL-31	11,13	12,12	10,10	13,13	16,18	6,9	X,X	8,11	10,10	-
0.78(28/36)	29	MOLT-16	11,12	12,12	12,12	9,10	16,16	9,9.3	X,X	8,11	11,12	-
0.78(28/36)	36	MOLT-17	11,12	12,12	12,12	9,10	16,16	9,9.3	X,X	8,11	11,12	-
0.78(28/36)	CRL-2914	M4A4	11,12	12,12	8,10	13,13	16,18	6,7	X,X	8,11	11,11	-
0.78(28/36)	CRL-2915	M4A4 GFP	11,12	12,12	8,10	13,13	16,18	6,7	X,X	8,11	11,11	-
0.78(28/36)	CRL-2916	M4A4 LM3-2 GFP	11,12	12,12	8,10	13,13	16,18	6,7	X,X	8,11	11,11	-
0.78(28/36)	CRL-2917	M4A4 LM3-4 CL16 GFP	11,12	12,12	8,10	13,13	16,18	6,7	X,X	8,11	11,11	-
0.78(28/36)	CRL-2918	NM2C5	11,12	12,12	8,10	13,13	16,18	6,7	X,X	8,11	11,11	-
